# Investigating the Performance of a Zinc Oxide Impregnated Polyvinyl Alcohol-Based Low-Cost Cation Exchange Membrane in Microbial Fuel Cells

**DOI:** 10.3390/membranes13010055

**Published:** 2023-01-02

**Authors:** Sunil Chauhan, Ankit Kumar, Soumya Pandit, Anusha Vempaty, Manoj Kumar, Bhim Sen Thapa, Nishant Rai, Shaik Gouse Peera

**Affiliations:** 1 Nanomaterials Lab, Department of Physics, School of Basic Sciences and Research, Sharda University, Greater Noida 201310, Uttar Pradesh, India; 2 Biopositive Lab, Department of Life Science, School of Basic Science and Research, Sharda University, Greater Noida 201306, Uttar Pradesh, India; 3 Department of Physics and Materials Science and Engineering, Jaypee Institute of Information Technology, Noida 201309, Uttar Pradesh, India; 4 Department of Biological Sciences, WEHR Life Sciences, Marquette University, Milwaukee, WI 53233, USA; 5 Department of Biotechnology, Graphic Era Deemed to be University, Dehradun 248002, Uttarakhand, India; 6 Department of Environmental Science, Keimyung University, Dalseo-gu, Daegu 42601, Republic of Korea

**Keywords:** heterostructure, polyvinyl alcohol (PVA), proton exchange membrane, microbial fuel cell, power density, proton conductivity

## Abstract

The current study investigated the development and application of lithium (Li)-doped zinc oxide (ZnO)-impregnated polyvinyl alcohol (PVA) proton exchange membrane separator in a single chambered microbial fuel cell (MFC). Physiochemical analysis was performed via FT-IR, XRD, TEM, and AC impedance analysis to characterize thus synthesized Li-doped ZnO. PVA-ZnO-Li with 2.0% Li incorporation showed higher power generation in MFC. Using coulombic efficiency and current density, the impact of oxygen crossing on the membrane cathode assembly (MCA) area was evaluated. Different amounts of Li were incorporated into the membrane to optimize its electrochemical behavior and to increase proton conductivity while reducing biofouling. When acetate wastewater was treated in MFC using a PVA-ZnO-Li-based MCA, the maximum power density of 6.3 W/m^3^ was achieved. These observations strongly support our hypothesis that PVA-ZnO-Li can be an efficient and affordable separator for MFC.

## 1. Introduction

Conventional energy supplies are a key contributor to greenhouse gas emissions, which have resulted in global warming and climatic change, prompting today’s substantial scientific studies into non-conventional renewable energy sources [1]. On the other hand, despite low treatment performance, most traditional filtration technologies need tremendous power to handle effluents produced by municipalities and industries. Governments, academics, and other officials worldwide have moved their emphasis to the study and application of alternative wastewater processing methods with lower energy demands and treatment performance [2,3]. Microbial fuel cells (MFCs) are a type of technology that not only harvests bioenergy but also serves as a bioremediation system by treating wastewater at the same time [4,5].

Exoelectrogenic bacteria are used in microbial fuel cells as biocatalysts to oxidize the organic compounds in sewage and produce electricity, which lowers the amount of energy needed for wastewater treatment processes. [6,7,8,9]. The cost of the proton exchange membrane (PEM) is one of the major concerns in applying MFC for bioremediation and bioenergy applications. Nafion, a polymer of tetrafluoro ethane, was first designed as a selective separator for protons in the chlor-alkali electrolyte in the late 1960s and has long been utilized as a PEM in MFCs. Despite its widespread use as a PEM, Nafion has several disadvantages, including a high price, protons that enable other cations to move and accumulate, biofouling, and sulfur toxicity [10]. This led researchers to work on an alternate yet cost effective PEM that can function effectively or equivalently and have a reduced negative influence on MFCs [11].

Owing to their ability to cross link, functional group and low-cost polymers such as polyvinyl alcohol (PVA) have been found to have strong hydrophilic characteristics and reasonable film-forming capabilities [12]. Moreover, compared to perfluoro sulfonic acid (PFSA)–based polymeric membranes, such as Nafion^®^, Flemion^®^, and Acipex^®^, and due to their physical and thermal features, they are suitable contenders for PEM [13,14]. Studies have extensively focused on the use of polymer ceramic composite membranes for use in MFCs, where hygroscopic metal oxide atoms, such as Al_2_O_3_, TiO_2_, and SiO_2,_ are applied as inorganic fillers to tremendously enhance the effectiveness of a polymer electrolyte to absorb water while also allowing MFC procedure at high temperatures and low relative humidity [15,16,17].

ZnO is a potential candidate material for research study because of its low manufacturing cost and physical features suited for various technical purposes. Solid oxide fuel cells (SOFCs) have large band gaps, which lead to poor electrical conductivities even at high temperatures (>300 °C) [18]. The most significant attribute of electrochemical devices is desired ionic permeability at ambient temperature and low charge transfer [19]. Other investigations have found that p-n heterojunction nanocomposite electrolytes allow solid oxide fuel cells to operate at low temperatures while maintaining enough ionic conductivity. ZnO-LCP (La/Pr doped CeO_2_) and BaCo.4Fe.4Zr.1Y.O3-δ-ZnO, for example, have ionic conductivity of 0.098 Scm^−1^ and 0.156 Scm^−1^ (at 500 °C), respectively [20,21,22]. Lithium (Li) is the best potential dopant molecule for SOFCs among the dopant atoms [23,24]. As reported in the literature, heat stability, electrochemical consistency, and small leakage flux are potential benefits of Li-based solid oxide fuel cells [25]. Additionally, the essential benefit of Li-doped oxide semiconductors for modern technological purposes is that they are ideal electrolyte materials for cold-temperature solid oxide fuel cells [24,26].

ZnO is a semiconductor with a large band gap and a natively n-type electrical carrier. Doping ZnO using transition metals and alkali metals, on the other hand, can result in p-type conductance. As a result, doping atoms alter electrical characteristics and ionic conductivity in ZnO. P-type conductors were reported for Li-doped ZnO semiconductors, and significant amounts of Li doping (16 mol percent doped ZnO) resulted in electrically insulated material [27]. Internal point gaps of Li replacements and intervening Li atoms were formed by the quantity of Li doped in ZnO, which changed the electronic and ionic conductance and calming technique as temperature increased. Li atoms may be easily absorbed into the ZnO lattice, and Li atoms diminish Zn vacancy defect quantities in the ZnO lattice, according to the literature [15,28]. Li doping resulted in significant cementation in a material, which increased ionic conductivity [26]. The development of p-n heterojunctions improves the ionic transportability of semiconductor materials. In particular, the researchers demonstrate that Li^+^ ions have strong ionic conductivities, particularly in glassy solid electrolytes [29,30,31].

MFCs potentially utilize biodegradable sources for power generation. MFC is a biochemically catalyzed process employing electrochemically active bacteria (EAB) capable of oxidizing biodegradable organic matter and transferring electrons to an electrode. MFC is gaining prominence as a viable alternative technology for capturing electricity through wastewater treatment, providing sustainable energy generation from a plentiful and low-cost source [32]. To make MFC economically feasible, manufacturing costs must be reduced by rationalizing its structure to obtain increased coulombic efficiency (CE) and volumetric power density [33]. Not long ago, good progress is seen in attempts to improve MFC performance. Although MFCs without membranes have been described and discovered to be promising due to their budget friendly nature, easy configuration, and relatively more power output on a small scale, major challenges associated with the absence of a membrane include increased substrate and oxygen diffusion, which lowers the bio electrocatalytic activity of the EAB and coulombic efficiency [34]. Given the challenges, there is a significant drive to create cost effective membranes, which are essential for expanding the use of MFCs and creating cost-effective treatment systems.

Recently, membranes of electrolyte polymers based on polyvinyl alcohol (PVA) have piqued the interest of researchers due to their wide range of applications in proton exchange, pervaporation, fuel cells, and so on [35]. Low-cost PVA-inorganic composite membranes have high thermal, dimensional, and mechanical stability and outstanding electrochemical and hydrophilic characteristics, all of which are required for an ion exchange membrane [36]. The inclusion of inorganic dopants improves the ion transport characteristic of polymeric membranes.

Recently, ZnO/PVA-based composite membranes have gained a lot of attention. Asadpour et al., 2022, explain the unique structural and functional characteristics of how ZnO/PVA membranes offer significant promise in a variety of electrical, optical, and water treatment applications [37]. Abdullah et al., 2021, found that ZnO-PVA membranes’ ionic conductivity has been examined over a wide frequency range and at various temperatures [38]. According to impedance analysis, the sample containing 2% ZnO-NPs had lower bulk resistance than undoped polymer electrolytes. A tiny amount of ZnO-NPs greatly improved the proton conductivity; the maximum attainable ionic conductivity at room temperature was 4.71 × 10^−4^ S/cm [38]. To assess how much ZnO-NP content affected the transport characteristics of the ready-made proton-conducting NSPEs, we employed the Rice–Roth model. The findings demonstrate that an increase in both the amount and mobility of proton ions is responsible for the observed enhancement in ionic conductivity. It is hypothesized that, due to its low cost and excellent performance, ZnO-NPs have tremendous potential for MFC applications with PVA membrane in large-scale wastewater treatment for effective substrate removal and high-power output [39]. It is also reported that ZnO NP has antimicrobial activities. Despite its impressive characteristics, no research has been performed using the MFC’s ZnO/PVA-based membrane. The present study synthesizes low-cost ZnO-NPs for PVA membrane in single-chambered MFCs as membrane cathode assembly (MCA). The improvement of the membrane structure can be further made by increasing its conductivity. One of the approaches is to increase electrical properties of ZnO. Electrical measurements reported by Majumdar et al. [40], hot probe measurements, and a positive Hall coefficient verify that all Li-doped nanocrystalline films are of the p-type [S]. Using the van der Pauw technique, the resistivity of the films was determined to be 4.35 × 10^−2^ Ohm-cm for the 5% Li-doped samples and 1.30 × 10^−2^ Ohm-cm for the 10% Li-doped samples. The carrier mobility and concentration were found to be 2.1 × 10^17^ cm^−3^ and 0.67 × 10^2^ cm^2^/Vs for 5% Li-doped ZnO and 3.3 × 10^17^ cm^−3^ and 1.47 × 10^2^ cm^2^/Vs for 10% Li-doped ZnO, respectively. The conductivity increases with increasing the Li content in ZnO nanocrystalline samples [41]. Nevertheless, no report was available on application of ZnO-NPs-doped PVA based membrane in MFCs.

In this work, the synthesis and characterization of ZnO-NPs-doped PVA and further Li doped ZnO NP were performed. The performance of ZnO-impregnated and Li doped ZnO-impregnated PVA-based low-cost CEMs was investigated in terms of volumetric power output and Coulombic efficiency for their application in MFC. The effect of ZnO NP concentration and Li-doped ZnO on the PVA membrane was evaluated. First the concentration of ZnO np was optimized in MFCs having PVA membrane. Further, Li was included in the optimized ZnO-PVA in order to increase membrane conductivity and overall performance. The ZnO-NPs-doped PVA performance is compared to those of Li-doped PVA in terms of power production, chemical oxygen demand (COD) elimination, and Coulombic efficiency.

## 2. Materials and Methods

### 2.1. Synthesis and Characterization of ZnO and Li-Doped ZnO

The cost-effective sol–gel method synthesized pure and Li^+^ doped ZnO nanocrystalline samples [42]. ZnO is typically prepared by dissolving zinc acetate (CH_3_COO)_2_ Zn(2H_2_O) in deionized water and then adding 60 mL of ethylene glycol monomethyl ether (C_3_H_8_O_2_). For eight hours, we agitated the dissolved transparent solution vigorously over a hot plate (60 °C). Therefore, the fluffy gel was obtained when the produced transparent solution was dried in an oven kept for 2 days at 120 °C. The ZnO nanocrystalline sample was obtained after calcining the fluffy gel at 350 °C for 20 h in air atmosphere. Similarly, Zn_1−x_Li_x_O nanostructured samples were also generated through the sol–gel method. Lithium nitrate was supplied to the precursor solution in an approximated amount as a dopant, and the rest of the synthesis process remained the same. In this method, calculated amounts of Zinc acetate and Lithium nitrate were used as the precursors and dissolved in the deionized water. Then, the calculated amount of the ethylene glycol monomethyl ether (C_3_H_8_O_2_) was further added to the transparent solution. The dissolved clear solution was heated to 60 °C and agitated vigorously for eight hours before being dried in an oven for 2 days at 120 °C. Finally, the fluffy gel was calcined at 350 °C for 20 h in air ambiance resulting in Zn_1−x_Li_x_O nanocrystalline samples. X-ray diffractometer (Shimadzu 6000 XRD, Kyoto, Japan) through CuKα radiation source (λ = 1.5406 Å) at 40 kilovolt and 30 mA was utilized to structurally characterize the ready samples. The Rietveld refinement of the XRD patterns to refine structural and microstructural parameters was performed using the software program FullProf.2 k (Version 4.30-April 2008-ILL JRC) suit package. The peaks are modeled in the refinement using the Thompson–Cox–Hastings (TCH) profile function with asymmetry [43]. This asymmetric TCH function separates the effects of strain broadening and particle size in the experimental profiles [44]. All possible instrumental modifications were taken into account when modelling the XRD experimental patterns of the synthesized samples against the Si reference sample. Simulation results are observed in this study after key microstructural and morphological parameters were fine-tuned, including lattice strain, particle size, and shape, etc. The peak parameters (U, V, and W Caglioti parameters) were obtained for the XRD instrument machine using a silicon standard sample and reserved unchanged during the refinement process. Each XRD pattern’s background is linearly interpolated between a set of background locations. Peaks in the XRD pattern are positioned by making a series of zero-error adjustments to the fitting procedure. Minimization and X-ray diffraction (XRD) patterns are inspected with the use of the reliability index parameters R_exp_ (expected error) and R_wp_ (weighted residual error).

Diffraction, particle size, and morphology pattern images were captured using a high-resolution transmission electron microscope (HRTEM, JEOL-2100F Tokyo, Japan) functioning at 200 kilovolts. The optical properties of the synthesized nanocrystalline samples were studied using Shimadzu UV-Vis-1800 spectrometer, Kyoto, Japan. Photoluminescence (PL) spectroscopy (PerkinElmer, LS-55, Woodbridge, ON, Canada) measurements of the samples of undoped and Li^1+^ doped ZnO nanocrystalline were executed with the excitation wavelength of 325–340 nm at room temperature.

### 2.2. Models and Calculation Methods

The subsequent models were assembled and studied: pristine ZnO supercell with 32 atoms Zn_16_O_16_, Zn_15_LiO_16_ supercell, Zn_16_Li_in_O_16_ supercell (one Li at interstitial position), and Zn_16_Li_in_O_15_ supercells (one Li and V_O_ one oxygen vacancy). The theoretical calculations were achieved with the generalized gradient approximation (GGA) as instigated in the PWSCF code of the Quantum ESPRESSO integrated package. The Perdew–Burke–Ernzerhof (PBE) scheme designated the exchange–correlation potential. In all the described systems, the GGA + U scheme has been used to correct the band gap energies, and the value of U for the Zn and O atoms are 10 eV and 7 eV, respectively. Zn-3d^10^ 4s^2^, O-2s^2^2p^4^, and Li-1s^2^ 2s^1^ valence electron configurations have been used to construct pseudopotentials. The cut-off energy of the used plane-wave expansion stayed 225 eV with the convergence accuracy of the geometric optimization and energy was 1.0 × 10^−6^ eV/atom. The Monkhorst–Pack 5 × 5 × 4 k-point mesh was exploited to estimate first Brillouin zone with a smearing of 0.01 Ry in all the systems.

### 2.3. Preparation of Membrane Cathode Assembly (MCA)

The cathode consisted of carbon felt (unless otherwise specified, round sheet has an effective diameter of 3 cm). To make ink with a catalyst of cathode, 0.01 mg/cm^2^ dust of MnO_2_ (20 wt% MnO_2_, Sigma-Aldrich, Mumbai, India) and 3 mg/cm^2^ carbon black Vulcan XC-72 from Cabot Corp of Boston, MA, USA are combined with aqueous solution of 1.2 L of PVA (1% *w/v*) as the binder at 1:1 acetone–isopropyl of 20 mL [45]. The PVA- MnO_2_ carbon black aqueous-acetone solution was ultrasonicated for 30 min and then utilized as ink to cover the cathode. On the carbon felt, ink containing a cathode catalyst was applied before being baked at 60°C. In this method, the membrane (ZnO-NPs PVA) is directly bonded to an adjustable carbon felt electrode doped with MnO_2_ catalyst at 85 °C hot pressing for 5 min; the membrane cathode assembly (MCA) was constructed (Moore Max Ton Hydraulic Press- 800 kPa, Mumbai, India) [46].

### 2.4. Microbial Fuel Cell Construction and Operation

Five identical single chambered MFCs of the cylindrical type were selected for the experiment with Borosil glass bottles of 500 mL, with a side aperture that is 3 cm in diameter, is attached at the bottom. Anode and cathode electrodes were spaced apart by 4 cm in the hot-pressed MCA (7.07 cm^2^ by 3 cm in diameter), which is made of PVA-ZnO with catalyst-loaded carbon felt. The MFC consists of an anode on one side and an MCA on the other. The carbon felt makes the 32 cm^2^ (4 * 4 cm^2^, both side exposed) anode electrodes free of catalysts. A comparative investigation was conducted utilizing two distinct membranes, native PVA-ZnO (M1) and PVA-ZnO-Li with 0.5 wt% of Li content (M3), in MFCs with the same design. The adequate anolyte volume was 350 mL. Investigations were also conducted into the effects of mixing various amounts of ZnO (0.5, 1.0, 1.5, 2.0 wt%) in PVA-ZnO-Li on the effectiveness of MFC tests.

MFC was employed for the oxygen mass transfer investigation and the biofouling experiment. The single-chambered MFC configuration had a 500 mL manufactured glass container with a bottom side aperture (28 cm^2^). PEM (PVA-ZnO-Li) was applied while the chamber was clamped. The cathode electrode was a platinum-impregnated tantalum electrode from Titanium Tantalum Products Ltd. Uttar Pradesh, in India, measuring 3 × 5 cm^2^. Carbon felt, measuring 6 × 4 cm^2^ as an anode electrode, was used.

### 2.5. Inoculum and Anolyte

Throughout the investigation, acetate (carbon source) containing synthetic wastewater was used. For all MFCs, the synthetic acetate wastewater’s COD ranged from 4.99 to 5.01 g/L [13]. The initial pH in each MFC was held constant at 7.0. Mixed consortia of anaerobes isolated from the septic tank bottom sludge of Sharda University Noida served as the inoculum. A 1-mm filter was used to separate the inoculum sludge, which was then warmed for 15 min at 100 °C before cooling. The mixed culture was enhanced with designed synthetic wastewater (DSW) and rinsed three times in saline buffer (at a speed of 5000 rpm) before being inoculated and incubated for 48 h at room temperature and 100 rpm. The enhanced culture that resulted from this was injected with feed at a concentration of 2.8 g VSS/L. Before starting the MFC in batch mode, pre-treated mixed microflora was introduced into the anodic compartment. There were several cycles run. The cycle period for each batch was 48 h. After each feeding, 2 min of oxygen-free N_2_ were used to sparge MFC to maintain an anaerobic microenvironment. Before being fed, the pH of the effluent was adjusted to a target value of 7.0.

### 2.6. Membrane Analysis

The membrane’s proton conductance was estimated using electrochemical impedance spectroscopy (EIS; ECLab SP 50/150, Bio-logic, Seyssinet-Pariset, France) in the frequency range of 100 kHz to 100 MHz [13]. With a two-chambered test unit composed of plexiglass equipped with a DO probe, the oxygen crossings were calculated in measures of the oxygen diffusion coefficient (Lutron DO-5509, Noida, India). The exact layout was used to evaluate the acetate diffusion coefficient. The density of diffused acetate was determined using a gas chromatograph, India equipped with a flame ionization detector (FID) and a DB-FFAP fused-silica capillary column using nitrogen as the carrier gas.

Calculation of water uptake was performed by measuring the membranes’ weight change after being submerged in water for 24 h, and the ions exchange capacity was calculated using the titrimetric technique [47]. Two Luggin capillary reference electrodes (CH Instruments, Inc., Bee Cave, TX, USA, RE-5B; +0.197 V vs. a standard hydrogen electrode, SHE) were used to quantify the ion transport rate in a two-chambered electro dialytic cell composed of plexiglass with circular test membrane (9.62 cm^2^ of the exposed surface) [47]. The tensile strength analysis was used to determine the mechanical properties of the manufactured membranes. Standard testing equipment (Tinius Olsen, Norway) scanned a 50 mm 10 mm piece of PEM at a scanning speed of 12.5 mm min^−1^ to determine its ultimate tensile strength and percentage of extension.

### 2.7. Analytical Measurements and Calculations: Electrochemical Evaluation of MFC

A data acquisition system (Agilent Technologies, Selangor, Malaysia) was used to record voltage across a constant external resistance (R_ext_) of 100. Current (I) flowing through the circuit was determined to be in amperes (A) using Ohm’s law (V = I._Rext_), where V is the OV measured. According to Equation (1), the MFCs’ total power output was assessed [48]:

(1) P=I×V

The power density curve displayed during polarization was used to calculate the maximum power density produced by MFCs by varying the external resistances from 10 to 10,000 (GEC05R Decade Resistance Box, Bengaluru, India). The slope frequency of the linear section of the voltage vs. current curve was used to determine the internal resistance. After standardizing with the anodic chamber operating volume (Vand, L) according to Equation (2), the evaluation of the volumetric power density (P, W/m^3^) was conducted.

(2) $$i_{d}=\frac{V_{\mathrm{and}}}{\mathrm{RA}};p_{d}=\frac{{V_{\mathrm{and}}}^{2}}{\mathrm{RA}}$$

### 2.8. Analytical Evaluation and Measurement

Both power production and coulombic efficiency were looked at while evaluating the functioning of the MFC. A detailed discussion of measurement and calculation techniques for calculating Coulombic efficiency, power density, current, and electrode potentials, is provided in the accompanying information (SI). A single chamber and sterile reactor that was mixed using a magnetic stirrer bar, the oxygen coefficient of mass transfer (kcm, cm/s) was measured. A partition with a ZnO-NPs PVA membrane, M3 (8 cm × 8 cm), was placed between the cathode and anode chambers of MFC. The cathode was filled with 4.33 g of Na_2_HPO_4,_ 2.69 g of NaH_2_PO_4_H_2_O, 0.13 g of KCl, 0.31 g of NH_4_Cl, and a 50 mM phosphate buffer solution in 1000 mL at pH 7. By using N_2_ gas to purge the anode chamber, the dissolved oxygen (DO) was reduced to zero prior the kcm measurement. The dissolved oxygen of the cathode chamber was kept saturated during the kcm measurement with a constant air supplied by an aquarium pump operating at 2 L/min. The data logger software was used to record the DO concentration at intervals of 15 min for a batch of 48 h. For each chamber’s liquid volume (V), membrane thickness (Lt), cross-sectional area (A), and batch time (t), we were able to determine the diffusion coefficient (DO, cm^2^/s) and the kinetic constant (kcm) using Equation (1). The oxygen transfer coefficient (kcm, cm/s) was derived using the formula:

(3) kcm=Dcm/Lt

Short circuit currents (Isc) were measured when the cathode and anode were directly attached to the multimeter. The multimeter used was a Precision Mastech Enterprises Co., Hong Kong, Mastech 6000, Kowloon, Hong Kong counts digital multimeter. The current interrupt approach was used to determine the MFCs’ internal resistance. After the MFC reached a stable state for its potential (V) and current output (I), the circuit was opened, causing the cell voltage (VR) to spike suddenly before rising more gradually.

Analyte chemical oxygen demand (COD) values were measured with a COD measuring kit (UV-1900i Spectrophotometer, SHIMADZU, Kyoto, Japan) in accordance with APHA standard procedures. A desktop pH meter was used to keep track of the pH levels. The membrane containing protein content was measured using the Lowry technique. The membrane was retrieved from MFC after 76 days of operation, maintained with 20 mM phosphate in an autoclave buffer, had debris stuck to it scraped off, and was suspended in a Falcon tube with 10 mL of lysis solution for 20 min to aid in protein extraction. After diluting an aliquot of the well-blended mixture with lysis buffer, the SDS solution was added and the whole thing was vortexed. A spectrophotometer was used to test the sample’s absorbance at a wavelength of 750 nm after 30 min.

### 2.9. Biofilm Formation Studies on ZnO-Impregnated PVA Membrane

The growth media for bacteria (acetate wastewater) was introduced for six batch cycles into the anode chamber of MFCs with ZnO-impregnated PVA membrane after the bacteria had been washed. Using five distinct ZnO-impregnated PVA membranes, we determined and visually compared the microbial cells attached on the membrane towards anode side for each area and average biovolume of cells (PVA as control and ZnO impregnated in PVA at increasing ratios (1.50, 1, 1.00, 0.75, and 0.50 mg/cm^2^)). Following the completion of each round of the MFC biofilm formation experiment, the anodes were removed and subsequently sliced into pieces measuring roughly 5 mm by 5 mm. A PBS solution containing 0.1 mg/mL, 3 mM propidium iodide (PI) and using PI (SRL, India) was prepared. The biofilms were incubated in the stain solution for 30 min in the dark till stained. The biofilm samples were viewed on a confocal laser scanning microscopy (CLSM; ZeissMeta510; Carl ZEISS, Inc., New York, NY, USA) with a Zeiss dry objective LCI Plan-Neofluar (×20 magnification and numerical aperture of 0.5). Ten different places on each surface were used to analyze the average biovolume of the biofouling. The same process as previously described was used for picture capture and processing. The images were analyzed with the image processing software COMSTAT in Matlab 6.5 (The Math Works, Inc., Natick, MA, USA) to determine the exact biovolume (m^3^/m^2^) in the biofouling layer. In each sample, 10 places on each membrane were randomly selected, examined under a microscope, and evaluated. The CLSM image stacks were 3-dimensionally rebuilt using the Imaris program (Imaris Bitplane, Zurich, Switzerland). The average biovolume of cells attached to the membrane (towards the anode-facing side) was computed and assessed for Li-doped ZnO-impregnated PVA membranes at various concentrations [49].

## 3. Results

### 3.1. XRD Rietveld Analysis

**Figure 1:**
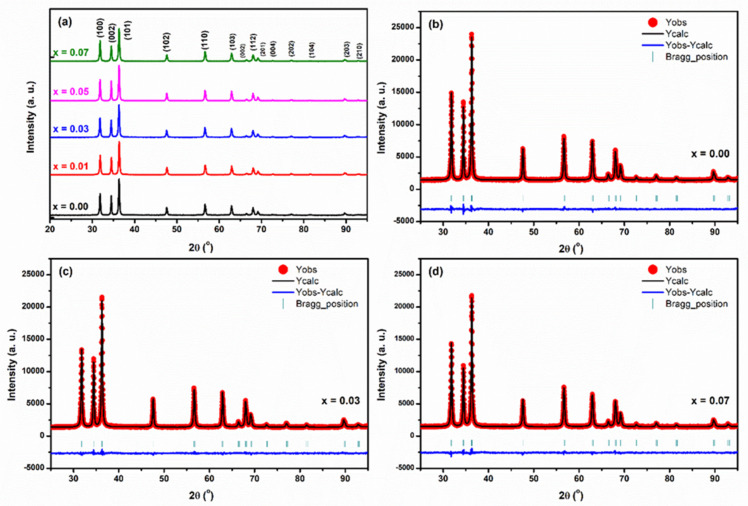
(**a**) XRD patterns of Zn_1−x_Li_x_O nanocrystalline samples calcined at 350 °C. Rietveld refined XRD data of Zn_1−x_Li_x_O nanocrystalline samples. (**b**) x = 0.00, (**c**) x = 0.03 and (**d**) x = 0.07.

Figure 1a shows the patterns of X-ray diffraction of Zn_1−x_Li_x_O nanocrystalline samples. Zn_1−x_Li_x_O samples are highly crystalline, and the diffraction patterns specify the development of hexagonal wurtzite crystal structure with the space group P63mc according to the JCPDS card No. 36-1451 [42]. As shown in Figure 1, the peak positions show a slight variation with the Li^1+^ substitution in ZnO crystalline samples related to the pristine ZnO nanocrystalline sample. ZnO and Li substituted ZnO samples have lattice constants that are consistent with those reported for pure ZnO, as estimated by the authors. The generated Li-doped ZnO nanocrystalline samples have had their phase purity, lattice parameters, and symmetry determined using Rietveld refinement of X-ray diffraction patterns in the space group P63mc (No. 186, Z = 2). Details of the refinement can be finest close-fitted Rietveld refined. X-ray diffraction patterns of the synthesized Zn_1−x_Li_x_O nanocrystalline sample are revealed in Figure 1b–d. In these XRD patterns, the places of the diffraction Bragg peaks contest fine with the described XRD pattern of nanocrystalline ZnO samples. No other phase consistent with metallic Zn, metallic Li, and the oxides of Li is realized within the XRD detection limit, as shown in Figure 1b–d, and the diffraction Bragg peak positions and background are well suited.

Consequently, the XRD profile refinement approves single phase formation of Zn_1−x_Li_x_O nanocrystalline samples in the wurtzite (P63mc symmetry) type hexagonal crystal structure and in-depth uniform distribution of Li^1+^ ions in ZnO nanocrystalline host lattice. The Rietveld refined structural lattice, atomic positional, and fitted reliability parameters are listed in Table 1. The ZnO lattice was found to have a preference for Li atoms, and this preference was confirmed by best-fitting calculations. It was established from a monotonic decrease in the lattice parameter ‘a’ (Å) and the unit cell volume V that the crystal structure of ZnO nanostructured samples is distorted as Li^1+^ concentration is increased, as seen by the Rietveld refinement of the XRD patterns (Å^3^). This distortion is due to the movement of the cations along the z-axis and is quantified by the amount by which the anion positional parameter u = [(1/(3(a/c)2)) + (1/4)] deviates from the optimal value of 0.375. The ionic position variable “z” coordinate u parameter of the oxygen atom is initiated to diminution with growing Li concentration in ZnO nanocrystalline samples. Thus, the distortions in bond angle and bond length were clarified. Due to differences in ionic radius and the monovalent state of the Li-ion compared to the divalent Zn-ion, Li doping in ZnO nanostructured crystals deformed the wurtzite structure.

Crystalline samples, whether they are strained, non-strained, or perfect, can be understood by referring to the BVS rule, which stands for the bond valence sum rule [44]. According to the (BVS) rule, the formal charge of the anion (cation) in a perfect crystal is always equal to the sum of the valences of the bonds surrounding the anion (cation). When sufficient degrees of freedom occur in the crystallographic structure, we find this rule to be effective in relieving the tension brought on by the presence of several, independently existing structural units. As a result, the deviation from the BVS rule can be used to quantify the strain between cation and anion bonds in the crystal structure. Table 1 lists the results of bond valence calculations for cations including Zn, Li, and anion O, where Zn-O and Li-O bond lengths are used. With an increase in Li content (from 1 to 7%), the BVS of Zn cation rises from 1.91 to 1.94 and eventually achieves the predicted value of 2. Increases in Li content in ZnO nanocrystalline samples result in a BVS of O that is lower than the predicted value of 2, falling from 1.91 to 1.85. These outcomes show that the Zn cations are underbonded, and the Li cations are over-bonded in this crystal structure; or because the Zn-O bonds are under tension and the Li-O bonds are under compression, the resulting structure is very metastable.

### 3.2. Analysis through Transmission Electron Microscopy (TEM)

**Figure 2:**
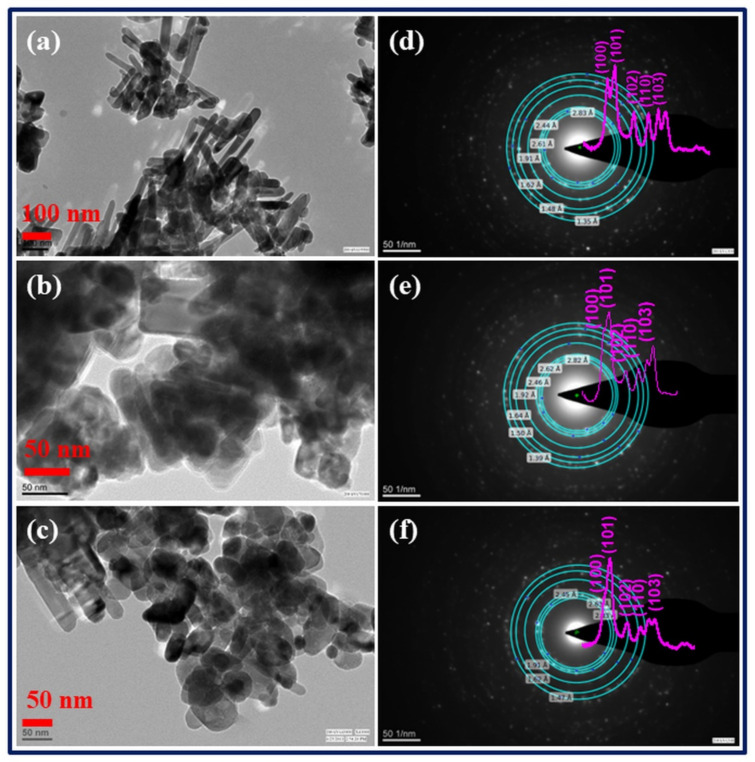
(**a**–**c**) TEM images of Zn_1−x_Li_x_O nanostructured samples (**a**) x = 0.00, (**b**) x = 0.03 and (**c**) x = 0.07. (**d**–**f**) SAED Pattern Zn_1−x_Li_x_O nanostructured samples, (**d**) x = 0.00, (**e**) x = 0.05 and (**f**) 0.07 sample.

The TEM method was used to characterize the crystallinity, shape, and size of the generated nanostructured samples, among other morphological and crystallographic properties. The low-resolution TEM images of Zn_(1−x)_Li_x_O: x= 0.0, 0.03, and 0.07 nanostructured materials are shown in Figure 2a–c, respectively. The morphology of pure ZnO samples is that of nanorods, with a diameter of 30 nm and a length of 500 nm; however, as the Li concentration in ZnO nanostructured materials is increased, the nanorods transform into hexagonal-spherical nanoparticles of size 30–50 nm. Additionally, the TEM pictures show that the nanostructured materials get bigger as the Li concentration rises in ZnO. Because of the induction of lattice strain, ionic size mismatches develop. Particle growth is slowed as a result of the system’s lattice stresses, which also improve the nucleation rate. Selected area electron diffraction (SAED), as shown in Figure 2d–f, is also used to investigate the polycrystalline nature of both undoped and Li-doped ZnO nanostructured samples. The SAED shows that polycrystalline samples have the distinctive ring pattern. The simulated ring pattern of SAED of Zn_1−x_Li_x_O nanostructured with the diffraction intensity of peaks is also displayed in Figure 2d–f. The SAED pattern is in great concurrence with the XRD pattern. The pink curves in Figure 2d–f represent the diffraction intensity ring profile of the selected area electron diffraction patterns using the tool “Diffraction Ring Profiler”. The electron diffraction pattern profiler was unable to resolve the closely spaced rings corresponding to the (110), (002), and (101) peaks, however, giving the right lattice spacing of 2.83 nm, 2.63 nm, and 2.44 nm for the ZnO nanorods. Due to the low resolution of the closely spaced electron diffraction rings, the electron diffraction ring intensity profile is slightly different. This shows the overlapping of the (110), (002), and (101) peaks.

**Figure 3:**
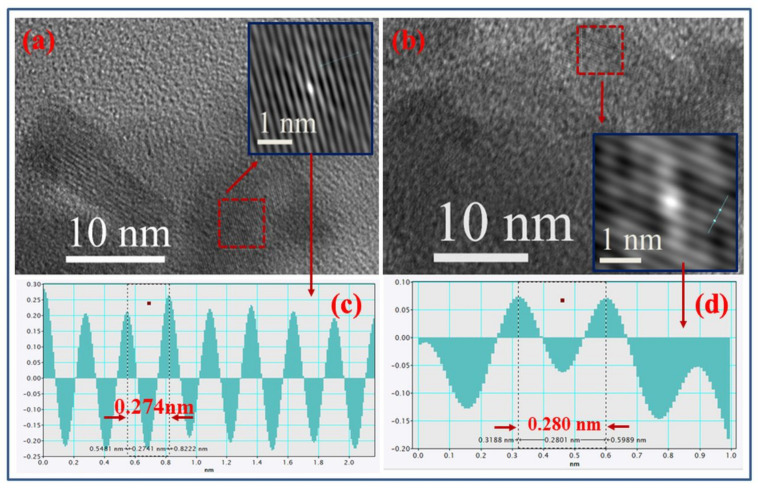
High-resolution TEM image of Zn_(1−x)_Li_x_O nanocrystalline samples (**a**) x = 0.0 and (**b**) x = 0.07 samples. The inset of (**a**,**b**) represents the autocorrelated images of the selected portion of the images. The intensity profile of the autocorrelated images used to measure the interplanar distance for (**c**) x = 0.0 corresponding to the (002) plane and (**d**) x = 0.07 corresponding to the plane (100).

The HRTEM images illustrate that the fine nanostructured samples were very considerably crystallized into single crystals by the specific interplanar spacing. In Figure 3a,b, we see a high-resolution TEM image of a specific region of the Zn _(1−x)_Li_x_O: x= 0.0, 0.07 samples. The interplanar spacing of the samples is ascertained from the intensity profile of the autocorrelated image of the selected portion by using the Gatan Microscopy Suite software. The autocorrelated images of the selected portion of the x = 0.00 and x = 0.07 samples are shown in the inset of Figure 3a,b. Figure 3c,d exhibit the lattice plane intensity profile pattern resulting in the inset of Figure 3a,b, confirming the interplanar spacing of about 0.274 nm and 0.28 nm, corresponding to the (002) and (100) planes of the ZnO and Zn_0.93_Li_0.03_O nanostructured samples, respectively.

### 3.3. UV-Visible Spectroscopy

**Figure 4:**
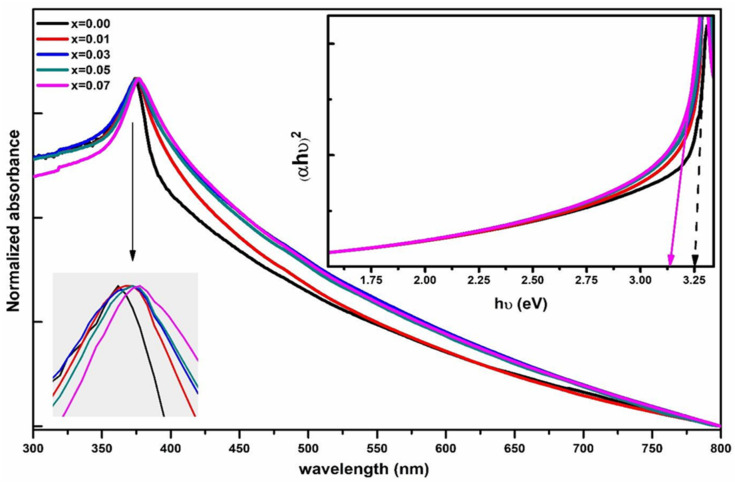
UV–visible absorbance spectra of Zn_1−x_Li_x_O nanostructured samples. Inset showed the Tauc plots of the absorbance spectra of Zn_1−x_Li_x_O nanostructured samples.

In Figure 4, we can see the UV–visible absorption spectra of Zn_1−x_Li_x_O nanostructured materials. The nanostructured sample powders were homogeneously dispersed in the ethanol with the help of the bath ultrasonicator for 50–60 min before recording UV–vis spectra. It can be seen in the inset of Figure 4 that the UV–vis absorption spectra of both undoped ZnO nanorods and 7% Li-doped ZnO nanostructured samples have excitonic peaks about 374 nm, which are offset from the intrinsic bandgap absorption peak. These absorbance peaks result from valence-to-conduction band (VB-CB) electron transitions (CB). The optical energy band gap of Zn_1−x_Li_x_O nanostructured samples has been calculated using the classical Tauc relation:

(4) $$\alpha h\upsilon =A{\left(h\upsilon -E_{g}\right)}^{n}$$

where A is a constant, and photon energy is hυ. In this case, the thickness of the cuvette is 1 cm; therefore, the energy band gap is E_g_, and the absorption coefficient is given by = (1/d) × ln(1/T), where T is the transmittance, and d is the absorption coefficient. The electronic transition that causes absorption determines whether n is 1/2, 3/2, 2, or 3. Direct transition is possible when n = 1/2, resulting in a direct band gap. The plots of (αhυ)^2^ versus hυ for Zn_1−x_Li_x_O nanostructured samples are shown in the inset of Figure 4. It is possible to obtain direct energy band gaps by extrapolating the linear portion of these figures to (αhυ)^2^ = 0. Undoped ZnO nanoparticles have a band gap of about 3.25 eV, which shrinks with increasing Li doping. The band gap of Zn_0.93_Li_0.07_O nanoparticles is around 3.13 eV. Band gap shirking due to Li^1+^ ion incorporation in ZnO nanoparticles can be seen when compared to those of undoped ZnO nanoparticles. The band gap energy can be lowered by incorporating Li^1+^ ions into the ZnO lattice, which can generate a shallow level inside the band gap. The DFT theoretical results also confirm the shallow levels inside the bandgap.

### 3.4. Analysis of Energy Bandgap Structure:

**Figure 5:**
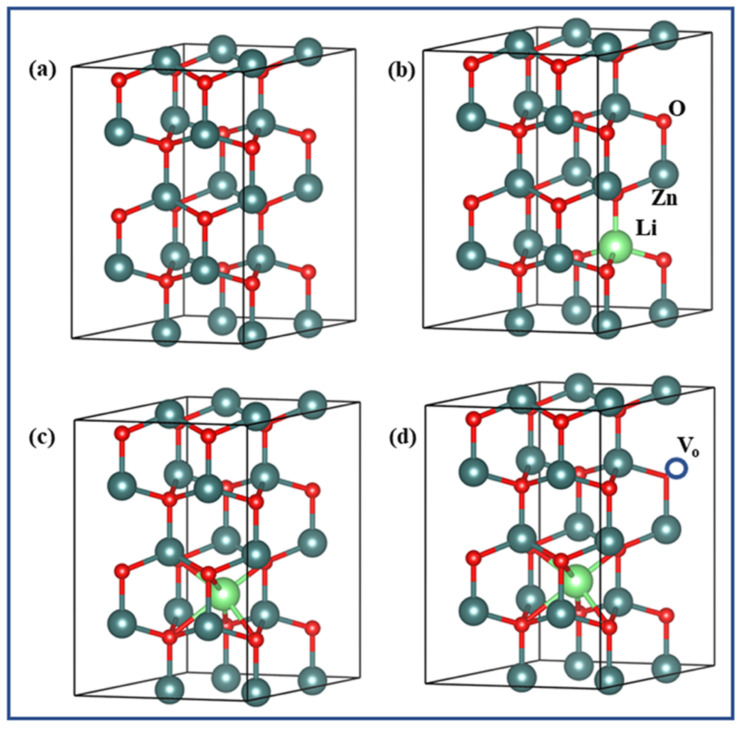
Researched ZnO models (Li atom-green ball, Zn atom-dark sky ball, and O atom-red ball): (**a**) Zn_16_O_16_, (**b**) Zn_15_LiO_16_, (**c**) Zn_16_Li_in_O_16_, and (**d**) Zn_16_Li_in_O_15_. V_o_ = oxygen vacancy.

**Figure 6:**
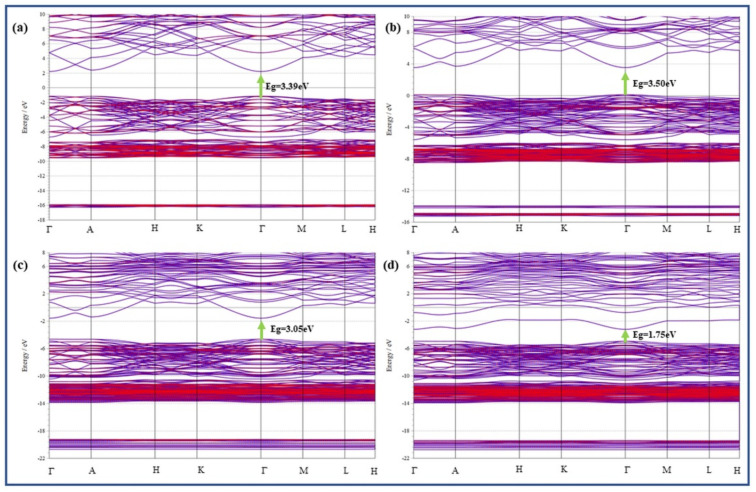
Band structures of (**a**) Zn_16_O_16_, (**b)** Zn_15_LiO_16_, (**c**) Zn_16_Li_in_O_16_ and (**d**) Zn_16_Li_in_O_15_.

The subsequent models were made: pure Zn_16_O_16_ supercell (2 × 2 × 2), a Li substituted ZnO lattice Zn_15_LiO_16_ supercell (one Li at Zn site), Zn_16_Li_in_O_16_ supercells (one Li interstitial Li_in_), Zn_16_Li_in_O_15_ supercells (one Li_in_ and one V_O_). The models studied have been shown in Figure 5a–d. Figure 6a–d demonstrate the energy band structures of ZnO, Zn_15_LiO_16_, Zn_16_Li_in_O_16_, and Zn_16_Li_in_O_15_, respectively.

Figure 6a displays the 3.39 eV band gap energy of pure ZnO, which is reliable by the experiment reported previously by our group. The outcomes of the theoretical investigations guarantee that the parameters chosen for this study were reasonable. The Fermi level shown in the Figure is at zero-point energy. Figure 6b–d show that the band gaps of Zn_15_LiO_16_, Zn_16_Li_in_O_16_ and Zn_16_Li_in_O_15_ are 3.50 eV, 3.05 eV and 2.75 eV, respectively. The findings indicate that, with the exception of the Li substituted ZnO: Zn_16_Li_in_O_16_, the band gaps of Zn_16_Li_in_O_16_ and Zn_16_Li_in_O_15_ systems are narrower than those of pure ZnO systems. Results from theory calculations are reasonably in line with those from experiments.

**Figure 7:**
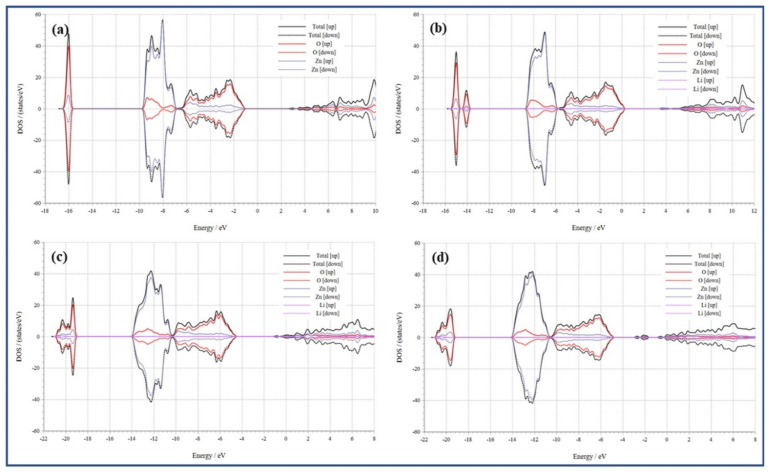
Density of states for (**a**) Zn_16_O_16_, (**b**) Zn_15_LiO_16_, (**c**) Zn_16_Li_in_O_16_, and (**d**) Zn_16_Li_in_O_15_.

The density of states (DOS) further explains the system of band gap change. Figure 7a–d displays the density of states (DOS) of pristine ZnO, Zn_15_LiO_15_, Zn_16_Li_in_O_16_, and Zn_16_Li_in_O_15_. The conduction band of pure ZnO is shown to be dominated by the Zn 4s, Zn 3p, and O 2p states in Figure 7a. The valence band is mostly provided by the O-2p state, Zn-3p state, and Zn-3d state, and the maximum of the valence band is primarily resolute through the Zn-4s state. The conduction band minimum is resolute through the Zn-4s state. Figure 7b–d displays that the conduction band of Zn_15_LiO_16_, Zn_15_Li_in_O_16,_ and Zn_15_Li_in_O is largely contributed via Zn-4s, Zn-3p, and O-2p states [50]. The Zn-4s state contributes to the lowest conduction band of Zn_15_LiO_16_, Zn_15_Li_in_O_16_, and Zn_15_Li_in_O_15_. The Zn-4s state contributes to Zn_15_Li_in_O_15_’s minimal conduction band [51]. The majority of the valence bands in Zn_15_Li_in_O_15_, Zn_15_Li_in_O_16_, and Zn_15_LiO_16_ are occupied by the Li-2s, Zn-3d, Zn-3p, Zn-4s, and O-2p states. The O-2p state mainly contributes the maximum valence band of Zn_15_LiO_16_, Zn_15_Li_in_O_16,_ and Zn_15_Li_in_O_15_. The DOS of Zn_15_LiO_16_, like pure ZnO, shows a symmetrical design, as illustrated in Figure 7b; thus, Zn_15_LiO_16_ is nonmagnetic. As a result, Zn_15_LiO_16_ does not produce an internal magnetic field. The Urbach tailing effect, brought about by hybrid coupling between Zn-4s and Li-2s states in the lowest conduction band, reduces the Zn_16_Li_in_O_15_ band gap. The maximal valence band is mostly unchanged in the meantime. In comparison to undoped ZnO, nonmagnetic Zn_15_Li_in_O_15_ has a greater band gap [52]. The improved hybrid coupling back-bond effect between Zn-4s, Zn-3p, and O-2p states at the minimal conduction band is responsible for this result, whereas the maximum valence band is essentially unaltered [53]. The energy level between the highest valence band and the minimum conduction band is split, and the band gap is shrunk due to the presence of an intrinsic magnetic field in Zn_15_Li_in_O_16_ and Zn_15_Li_in_O_15_.

### 3.5. Photoluminescence Spectroscopy

**Figure 8:**
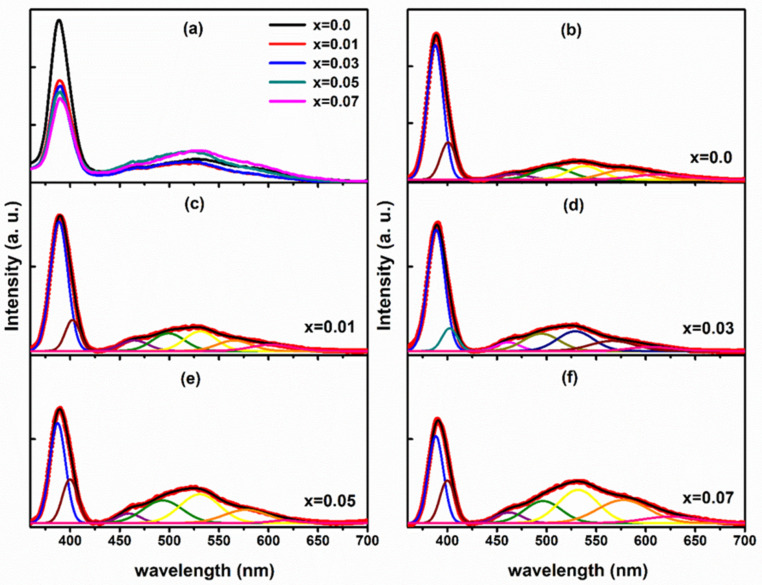
(**a**) Photoluminescence spectra of Zn_1−x_Li_x_O nanostructured samples. (**b**–**f**) Deconvoluted photoluminescence spectra of Zn_1−x_Li_x_O nanostructured samples.

At ambient temperature, the photoluminescence PL spectra were obtained in the 350–700nm region (Figure 8a), allowing us to investigate the defect-associated emission and the relative fluctuations of the defects in ZnO nanocrystalline samples due to the Li doping. Then, the PL spectra for Zn_1−x_Li_x_O nanostructured samples were deconvoluted using the peak-fit software with the Gaussian function. Figure 8a shows the PL spectra of Zn_1−x_Li_x_O nanostructured samples at room temperature. Direct recombination of the free exciton causes the UV emission at 385 nm, and the intensity of this emission is significantly greater than that of the visible luminescence. UV emission exhibits a red shift toward longer wavelengths when Li concentration increases in ZnO nanostructured samples, as seen by a small shift of peak energies toward longer wavelengths. The shifting shows a decrease in bandgap with increasing Li content. Acceptor-doped ZnO typically exhibits a narrowing bandgap, while donor-doped n-type ZnO shows a widening band gap. In detail, ZnO nanostructured samples doped with donor ions displayed a blue shift in the UV emission peak of photoluminescence spectra associated with pristine ZnO nanocrystalline samples. The detected red shift in the ultraviolet emission peak in Li-doped ZnO nanocrystalline samples may be an outcome of getting p-type ZnO nanocrystalline samples. The electrical transport measurements completed by Shakti et al. confirmed the p-type carriers in Li-doped ZnO nanorods. In the visible part of the photoluminescence spectrum, between 440 and 725 nm, the luminescence is so faint that it is scarcely detectable. Broad luminescence pattern in the visible region showed relatively bright green (530 nm) with faint blue emission, yellow luminescence (580 nm), and high orange-red emission (590–700 nm), all attributable to the different defect states present in the pure and Li doped ZnO nanostructured materials (460 nm) [41]. Impurities and imperfections in the interstitial state of negatively charged oxygen ions are depicted by the visible light emission [54,55]. In Figure 8b, the peak corresponding to 520 to 540 nm should arouse by the intrinsic defect of antistites oxygen OZn (2.38 eV) and interstitial oxygen Oi (2.29 eV).

In ZnO nanocrystalline samples, the presence of the visible luminescence bands is currently being discussed. Zn ions at the interstitial (Zn_in_) site are responsible for the violet-blue emission band at 460 nm. Due to the charge transfer process between the metal ion and the defect sites, the weak blue emission observed in Li-doped ZnO nanocrystalline samples should originate from the expanded Zn interstitial defect. Li^1+^ integration into ZnO nanocrystalline samples has been recently reported to induce green emission via oxygen vacancy (V_O_). As a result of the recombination of a hole with singly ionized V_O_ defects, it has been postulated that the green band emission observed in the ZnO nanocrystalline sample is caused by these defects. A result of their high activation energy, Li ions cannot replace O in the crystal lattice. It is possible for Li ions to permeate into the interstices between the lattices (Li_in_). As a consequence, Li^1+^ ions can form procedure substitutional along with interstitial point defects in the lattice of ZnO; consequently, to preserve the charge balance of ZnO lattice, intrinsic point defects of O_in_ and V_O_ are formed and co-occur in ZnO nanocrystalline samples. Table 2 shows the conductivity of the PVA-ZnO membrane increases with an increase in the concentration of Li-doping to a certain point. It shows the increment in conductivity till 1.50% (*w/w*). After increasing the concentration of ZnO-NP doping beyond 1.5%, no increment in conductivity has been observed in the MFCs.

### 3.6. Performance of PVA-ZnO-Li Membrane in MFC and Oxygen Mass Transfer

Using MFC reactors devoid of any inoculum, the oxygen crossing to the anode from the cathode via the ZnO-PVA membrane was evaluated. The medium solution in the MFC has previously been sparged with nitrogen gas to eliminate dissolved oxygen. The DO probe was placed on top of the MFC to collect data on the DO buildup for 10 h in a sterile medium solution in the anode. Contrarily, in this investigation, the DO concentration rose from 0.1 to 0.37 during 8 h in the case of ZnO-PVA membrane; K cm = 1.4 × 10^−6^ cm/s and D cm = 5.6 × 10^−8^ cm^2^/s were found in the MFC with PVA-ZnO-Li. Table 3 gives the comparison of the electrochemical performance of various types of ion exchange membranes in MFCs.

### 3.7. Voltage Generation

All five single chambered MFCs required two weeks to stabilize under a working condition of batch mode and 48 h of feed cycle time. A gradual rise in current was seen as operating time increased. On the sixteenth day for all sMFCs, the current generation achieved its highest value of 1.76 ± 0.4 mA at the external load of 100 Ω. Seven times in a row, acetate medium was transferred into the anode chamber of the mediator-free MFC, due to which stable power was produced.

**Figure 9:**
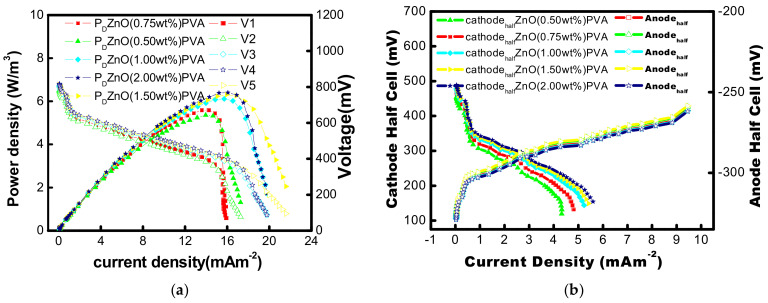
(**a**) The polarization curve of MFCs loaded with varying concentrations of Li-ZnO (0.50, 0.75, 1.00, 1.5, and 2.0 wt%). On the graph, the data points for voltage and power density are illustrated as solid and open symbols, respectively; (**b**) Half-cell polarization plots of MFCs with various (0.50, 0.75, 1.00, 1.5, and 2.0 wt%)-Li-ZnO concentrations. On the graph, the data points for the cathode half-cell and anode half-cell are shown as solid and open symbols, respectively.

Utilizing a variable resistance box with a resistance range of 90k to 10k and measuring the resulting voltage drop, the external resistance of the closed circuit may be adjusted, and polarization curves were produced after nine batch cycles. A total of 15 to 20 min were needed to obtain a steady reading. 1.5% wt ZnO-PVA provided highest volumetric power densities of 6.3 W/m^3^ according to the power densities measured. PVA without ZnO nanoparticles (as negative control) reached maximum volumetric power densities of 0.80 W/m^3^, which were 25% and 20% less than ZnO-PVA membrane-based MFC (Figure 9a,b). These findings unequivocally demonstrate that ZnO-PVA demonstrated superior power production. This was explained by ZnO-PVA having an order of magnitude less oxygen permeability. The effectiveness of MFC without ZnO NP in PVA membrane is decreased by the diffusion of oxygen from the cathode chamber to the anode chamber via the separator because some of the substrates are consumed directly by the oxygen instead of transmitting the electrons through the electrode and the circuit with PVA. With the application of various ZnO-NP loaded PVA membrane during the current work, the stable half cells (anode and cathode) are calculated. ZnO nanoparticle loads of 0.50, 0.75, 1.0, 1.5, and 2.00 wt% ZnO nanoparticles are used to observe the effect of ZnO nanoparticle load on the generation of power to PVA in MFCs. The wide variation in the potential of the cathodic half-cell of the microbial fuel cell is listed with different catalyst loads of ZnO nanoparticles in the PVA membrane (Figure 9b). The maximum volumetric power density (P_d,max_) of 6.3 w/m^3^ was observed for 1.50 wt% ZnO-Li. The Pdmax increases to 5.5, 6.0, and 6.2 w/m^3^ when the concentration of ZnO-Li was increased to 0.75, 1.0, and 1.5 wt% respectively. When the concentration of ZnO-Li is further increased from 1.5 to 2.00 wt%, P_d_ max was found almost unchanged (around 6.2 w/m^3^). So, 1.5%wt was found optimum for maximum power output. However, increasing the ZnO nanoparticle concentration from 0 to 0.25 wt% results in a twofold improvement of P_d,max_.

### 3.8. Effect of Li Addition in PVA-ZnO Membrane

The voltage generated in MFCs with PVA-ZnO-Li membrane was observed to research the impact of Li alliance on the general MFC activity in terms of power output and coulombic efficiency, where Li concentrations ranged from 0.0 wt% to 0.9 wt%. It was shown that the performance of MFCs can be considerably impacted by the amount of Li in the membrane (Table 2). The maximum power density of the MFC using the PVA-ZnO membrane without Li was 6.3 W/m^3^. The maximum volumetric power density increased with an increase in the concentration of Li from 0.0 to 0.3wt%. Additionally, increasing the amount of Li from 0.0 to 0.3 wt% increased coulombic efficiency by 15%. Due to its greater conductivity than the lower concentrations of Li in PVA-ZnO-Li membranes, the 0.3 wt% Li-loaded PVA-ZnO-Li membrane generates more power. The findings indicated that Li addition has a sizable impact on coulombic efficiency. However, when the concentration was further increased from 0.3 wt% to 0.9 wt%, the maximum volumetric power density was shown to decrease. The lower transmembrane potential loss may cause the MFC’s poor performance with the PVA-ZnO membrane without Li or Li at a very high concentration. The positive charge of Li in 0.9 wt% Li on ZnO PVA CEM may affect cation transfer owing to electrostatic repulsion.

Additionally, the concentration of biofouling (microbial cell) on the surface of PVA-ZnO membranes was found greater in Li contents (0.3 wt%) and was much lower than native PVA-ZnO membranes. According to previous research, the buildup of these bacteria and biofoulants causes a thick biofilm to grow on the surface of the CEM, increasing electrical resistance. Most foulants travel toward ion exchange membranes due to their surface charges and electric motilities. Liu et al. demonstrated that the cells lose integrity due to flattening after exposure to Li dispersions. Li has the propensity to cause bacterial cells to experience membrane stress, which causes the cellular structures to be destroyed. Reactive oxygen species (ROS) mediated oxidative stress, for example, was one type of oxidative stress claimed to be mediated by graphene-based materials. With the addition of Li on the PVA-ZnO membrane, less bio-foulant (bacterial cells) was discovered on the anode-facing side of the membrane. Overall, the results show that at an optimum level, the Li blending in the ZnO PVA membrane improved conductivity and decreased biofouling. Table 4 gives the comparison of the CEMs in different type of MFCs.

### 3.9. Electrochemical Impedance Spectroscopy Studies (EIS)

**Figure 10:**
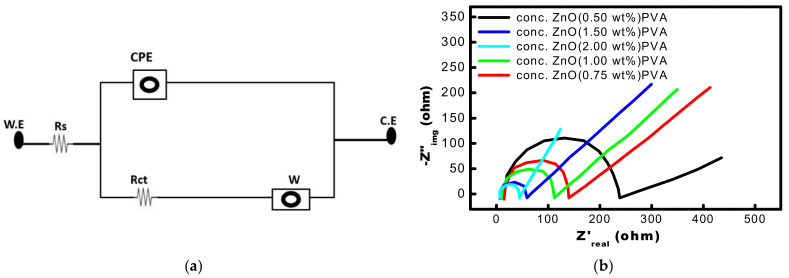
(**a**) The analogous circuit for a single-chambered MFC simulates different resistances. (**b**) Nyquist plot MFCs loaded with varying concentrations of Li-ZnO (0.50, 0.75, 1.00, 1.5, and 2.0 wt%) recorded at a frequency range of 1 MHz to 100 kHz (WE = working electrode, Rs = solution resistance, CPE = constant phase element, Rct = Charge transfer resistance, W = Warburg element, CE = counter electrode).

EIS is primarily used to evaluate electrode material quality, biofilm formation, and chemical reaction kinetics. Nyquist plots are used to investigate the electrochemical properties at the electrode interfacial layer (imaginary impedance vs. actual impedance). Internal losses such as diffusion transfer resistance, charge transfer resistance, and ohmic resistance are studied using EIS. This method employed a potentiostat (BioLogic SP150, Seyssinet-Pariset, France), controlled by EC-Lab software, Bio-Logic Seyssinet-Pariset, France to assess a broad frequency range (1 MHz to 100kHz). On Nyquist or Bode graphs, the outcomes of this method are shown. Figure 10a shows an equivalent circuit of MFCs.

The Nyquist plot contrasts original and fictitious impedance. The impedance at various frequencies is represented on the graph (Figure 10b). They conceal the frequency used to record a certain moment, which is a big problem. The Y-axis displays the phase angle and absolute impedance values, while the X-axis plots impedance as a function of frequency. In MFCs, the internal resistance of the system may be expressed as a clearly defined semicircle in the high-frequency region followed by a straight line in the lower-frequency range. The resulting resistance is greatest at the highest frequency point. The diameter of the semicircles may be utilized to calculate the resistance of charge transfer of each electrode (Rct). The Rct value is directly related to the catalyst and reactant or electrolyte interfacial contacts. MFC with a 2.00 wt% ZnO had a lower internal resistance (42.7 Ω), 1.50 wt% ZnO concentration had (47.5 Ω) than MFC with a 0.50 wt% ZnO concentration (223.611 Ω). Like this, as shown in Figure 10, the MFCs with ZnO concentrations of 0.75 wt% and 1.00 wt%, have shown 125 Ω and 102.78 Ω, respectively. There was no significant difference found in MFCs having 1.5% and 2.00 wt% ZnO impregnated PVA membrane.

**Figure 11:**
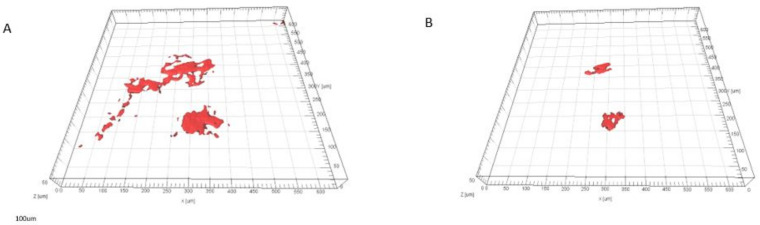
IMARIS 3-D images showing biofilms on PVA covered with Li-ZnO. (**A**) PVA with ZnO and (**B**) 0.3 wt% Li-ZnO-impregnated PVA. Dead cells can be seen as red clumps. Every image is a perspective that measures several 600 m by 600 m.

The components on the various PVA membrane surfaces and the biofilm formation were characterized using CLSM. The quantified results of biofilm formed on 1.5wt% of ZnO-impregnated PVA membranes in MFC experiments are shown in Figure 11.

The COMSTAT software was utilized to evaluate the data shown in Figure 11. Particularly, the minimum cell biomass was detected on the 0.3 wt% Li-ZnO-impregnated PVA compared to the ZnO-impregnated PVA. The minimum and maximum biovolume of the cell of 3.61 μm^3^/μm^2^ (±0.84) and 8.62 μm^3^/μm^2^ (±0.44) with 0.3 wt% Li-ZnO impregnated PVA and ZnO-PVA without Li, respectively. IMARIS 3D graphics verify COMSTAT’s findings. On 1.5 mg/cm^2^ ZnO-impregnated PVA, IMARIS shows less cell biomass.

## 4. Conclusions

In this study, a ZnO-impregnated and Li-doped ZnO-impregnated membrane was used, and different amounts of ZnO were added to PVA membrane of the MFC to improve its electrochemical behavior and increase its proton conductivity. Different proton exchange materials (ZnO NP as ion carrier) were impregnated in the PVA matrix using different concentrations of Li-ZnO (0.5, 0.75, 1.00, 1.5, and 2.00 wt%) as low-cost proton exchangers for MFC applications. Hygroscopic oxides, including ZnO, boost proton transfer because they develop channels via CEM’s microstructural (p-n heterostructure) changes brought on by Li-doping. When Li-ZnO np was impregnated over the PVA membrane, it was observed that the efficiency of current generation increased and was maximum when the concentration of Li-ZnO np was 0.3 wt%. These opened up the possibility of doing more research to reduce the substrate and oxygen crossovers rate, which negatively impacts MFC efficiency. The diffusion coefficient for dissolved oxygen was also calculated for the Li-ZnO-PVA membrane, which showed that the Li-ZnO-PVA membrane can be considered as an alternative to conventional PEMs. It may be concluded that the Li-ZnO-impregnated PVA-based PEM is ideal for potential implementation in MFC due to its characteristics and its low synthesis cost, demonstrating its viability for use in practical MFC systems.

## Figures and Tables

**Table 1:** By using Rietveld refinement to the XRD patterns, the crystal structural characteristics of Zn_1−x_Li_x_O nanoparticles were calculated.

Parameters	Symbol	x = 0.0	x = 0.01	x = 0.03	x = 0.05	x = 0.07
Lattice Parameters with Space Group P6_3_mc	a (Å) c (Å) V (Å^3^)	3.2482(1) 5.2034(25) 47.546(1)	3.2495(3) 5.2058(1) 47.605(1)	3.2488(2) 5.2048(1) 47.577(1)	3.2482(3) 5.2028(1) 47.540(1)	3.2477(2) 5.2038(1) 47.536(1)
Zn/Li at 2b (1/3, 2/3, 0) O at 2b (1/3, 2/3, u)	U	0.3798(5)	0.3808(4)	0.3808(5)	0.3796(5)	0.3085(5)
Bond Lengths (Å)	O-Zn/Li O-Zn/Li	1.976(3) 1.977(1)	1.976(1) 1.982(2)	1.975(1) 1.982(3)	1.975(1) 1.977(1)	1.975(1) 1.980(3)
Bond Angles (degree)	O-Zn/Li-O O-Zn/Li-O	108.44 (16) 110.48(3)	108.30(2) 110.62(3)	108.30(16) 110.61(3)	108.47(16) 110.45(3)	108.35(16) 110.57(3)
BVS	Zn	1.914(4)	1.909(4)	1.911(4)	1.914(4)	1.914(4)
	Li	-	1.004(1)	1.005(2)	1.006(2)	1.006(2)
	O	1.914(4)	1.900(4)	1.884(4)	1.869(4)	1.851(4)
Particle Size (nm)	D	30	28	34	48	-
Rietveld Reliability Parameters	R_P_ R_wp_ χ^2^	2.33 3.02 1.92	2.08 2.68 1.49	2.16 2.72 1.49	2.36 2.97 1.86	2.22 2.82 1.66

**Table 2:** Thickness, ionic conductivity, IEC, and water uptake capacity of different ZnO-PVA membranes.

Membrane	Thickness (µm)	Ionic Conductivity (S cm^−1^)	IEC (milliequivalent m^−1^)	Water Uptake (gm gm^−1^)
0.50% (*w/w*) PVA-ZnO	102	1.1 × 10^−2^	0.58	0.76
0.75% (*w/w*) PVA-ZnO	108	1.5 × 10^−2^	0.72	0.84
1.00% (*w/w*) PVA-ZnO	110	2.2 × 10^−2^	0.80	1.08
1.50% (*w/w*) PVA-ZnO	114	2.4 × 10^−2^	0.87	2.13
2.00% (*w/w*) PVA-ZnO	115	2.3 × 10^−3^	0.84	1.68

**Table 3:** Comparison of electrochemical performance of various types of ion exchange membranes.

Membrane	Type of Reactor	Volume of Anolyte (mL)	Anode (cm^2^)	Cathode (cm^2^)	Power Output (W/m^3^)	References
AMI-7001	Single chambered	-	228/Carbon cloth 228	carbon cloth/228 loaded with 0.5 mg/cm^2^ Pt/C	4.9	[56]
Nanoparticle Fe_3_O_4_ loaded	Dual chambered	760	20/Graphite plate	Graphite plate/20	0.052	[57]
Sulfonated poly in poly CEM	Air cathode	42.5	15/carbon paper sheet	carbon paper sheet loaded with 0.3 mg/cm^2^ Pt/C	6	[58]
Poly [2,5-benzimidazole] (ABPBI) CEM	Dual chambered	340	(0.5 mg/cm^2^ Pt/C loaded carbon) Graphite felt/36	Graphite felt/36 loaded with 0.5 mg/cm^2^ Pt/C	1.027	[59]
Li-ZnO-PVA CEM	Single chambered	110	Carbon felt (32 cm^2^)	Carbon felt (32 cm^2^)	6.3	This study

**Table 4:** Comparison of performance between CEMs used in the present study and prior studies.

MFC Type	PEM Material	Power Density	Reference
Dual chamber	Clay material	12.8 mW/m^2^	[56]
Two-chamber	Salt-bridge	78.2 mW/m^2^	[57]
Two-chamber	Ultrex	29.2 mW/m^2^	[58]
Dual chamber	TiO2-Si-impregnated PVA	247 mW/m^2^	[59]
Single chamber	Li-ZnO-PVA (0.9 wt%)	5.5 W/m^3^	This study
Single chamber	Li-ZnO-PVA (0.30 wt%)	6.6 W/m^3^	This study
Single chamber	Li-ZnO-PVA (0.00 wt%)	6.3 W/m^3^	This study

## Data Availability

Not applicable.
